# Emerging Therapeutic Biomarkers in Endometrial Cancer

**DOI:** 10.1155/2013/130362

**Published:** 2013-06-11

**Authors:** Peixin Dong, Masanori Kaneuchi, Yosuke Konno, Hidemichi Watari, Satoko Sudo, Noriaki Sakuragi

**Affiliations:** ^1^Department of Women's Health Educational System, Hokkaido University School of Medicine, Hokkaido University, N15, W7, Sapporo 060-8638, Japan; ^2^Department of Gynecology, Hokkaido University School of Medicine, Hokkaido University, N15, W7, Sapporo 060-8638, Japan

## Abstract

Although clinical trials of molecular therapies targeting critical biomarkers (mTOR, epidermal growth factor receptor/epidermal growth factor receptor 2, and vascular endothelial growth factor) in endometrial cancer show modest effects, there are still challenges that might remain regarding primary/acquired drug resistance and unexpected side effects on normal tissues. New studies that aim to target both genetic and epigenetic alterations (noncoding microRNA) underlying malignant properties of tumor cells and to specifically attack tumor cells using cell surface markers overexpressed in tumor tissue are emerging. More importantly, strategies that disrupt the cancer stem cell/epithelial-mesenchymal transition-dependent signals and reactivate antitumor immune responses would bring new hope for complete elimination of all cell compartments in endometrial cancer. We briefly review the current status of molecular therapies tested in clinical trials and mainly discuss the potential therapeutic candidates that are possibly used to develop more effective and specific therapies against endometrial cancer progression and metastasis.

## 1. Introduction

Endometrial cancer (EC) is the most common gynecological malignancy among women worldwide with 287000 new cases and estimated 74000 deaths per year [1]. 

EC has been dichotomized into two types with distinct underlying molecular profiling, histopathology and clinical behavior: less aggressive type I and highly aggressive type II. Most ECs are type I (approximately 75%) and are estrogen-dependent adenocarcinomas with endometrioid morphology [2]. They are usually diagnosed at an early stage and have a good prognosis (a 5-year survival rate of 80–85%) after surgery [2, 3]. In contrast, type II ECs with poorly differentiated endometrioid and serous histology are associated with myometrial invasion, extrauterine spread, and a lower 5-year survival rate (35%) [3–6]. Although patients with advanced or recurrent disease typically receive adjuvant chemotherapy and radiation, they have an extremely poor prognosis. A potential strategy for the treatment of these cases is to target EC cells by blocking key signaling pathways that are necessary for tumor development. 

## 2. Therapeutic Targets for EC

**Figure 1:**
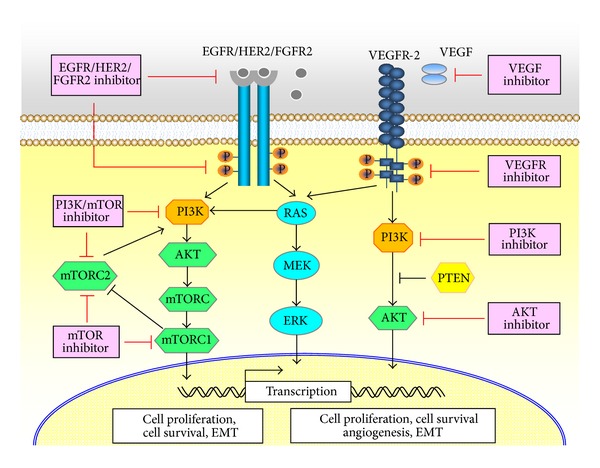
Therapeutic molecular targets for endometrial cancer. Type I endometrial cancer (EC) frequently exhibits altered PI3K/PTEN/AKT/mTOR signal pathway, whereas type II EC frequently shows mutations in p53 and HER-2 overexpression. The upregulation of EGFR and VEGF, dysregulated microRNAs, and activation of cancer stem cell (CSC)/epithelial-mesenchymal transition (EMT) programs are involved in oncogenesis and progression of both cancer types. Currently, clinical trials assessing the efficacy of mTOR inhibitor, EGFR/HER2 inhibitor, and antiangiogenic agent for EC have been conducted and demonstrated modest effects.

Type I EC frequently exhibits altered PI3K/PTEN/AKT/mTOR signal pathway [7–11]. Type II cancer predominantly shows mutations in p53 [12] and epidermal growth factor receptor 2 (HER-2) overexpression [13]. The upregulation of epidermal growth factor receptor (EGFR) [14, 15] and vascular endothelial growth factor (VEGF) [16], dysregulated microRNA (miRNA) [17], and activation of cancer stem cell (CSC)/epithelial-mesenchymal transition (EMT) programs are involved in oncogenesis and progression of both cancer types [18–20]. Owing to the high-frequency activation of PI3K/AKT/mTOR, EGFR/HER2 and VEGF-related pathway and their important roles in promoting EC growth and metastasis, new drug targeting these signals would be valuable to a very large number of patients with EC. Recently, clinical trials assessing the efficacy of mTOR inhibitor, EGFR/HER2 inhibitor, and antiangiogenic agent for EC have been conducted and demonstrated modest effects [21, 22] (Figure 1).

## 3. Challenges in the Molecular Therapeutics of Human Tumor

**Figure 2:**
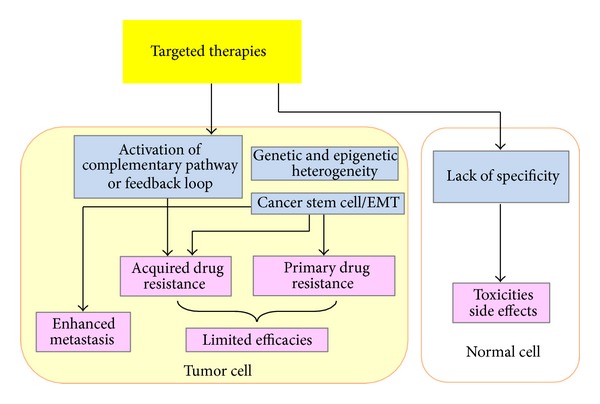
Challenges in the molecular therapeutics of human tumor. The clinical success of targeted drugs has been limited by key challenges, including primary/acquired drug resistance and unexpected side effects on normal tissues due to nonspecificity. The most frequent mechanisms of primary resistance are genetic/epigenetic heterogeneity and the existence of cancer stem cell. Acquired resistance can be caused by the secondary mutation in the target gene, activation of alternative pathway or feedback loop, and induction of EMT. Treatment of tumor cells with antiangiogenic agents can lead to a more hypoxic tumor microenvironment and enhance tumor cell invasion and metastasis by inducing the EMT- and cancer-stem-cell-like phenotype.

Although the therapeutic potential of targeted drugs for the treatment of human tumors appears promising, the clinical success of such drugs has been limited by key challenges, including primary/acquired drug resistance [23–25] and unexpected side effects on normal tissues due to nonspecificity [26] (Figure 2).

A portion of patients unfortunately do not respond to targeted agents (primary resistance), and the remainder might eventually acquire the resistance to targeted therapy despite an initial response. Various mechanisms of resistance have begun to be elucidated. The most frequently reported mechanism of primary resistance is genetic heterogeneity. For example, mechanisms of resistance to EGFR inhibitors are involved in point mutations, deletions, and amplifications of genomic areas of EGFR [23]. In addition to genetic alteration, epigenetic changes, such as DNA methylation at CpG islands, have been linked to the development of resistance to multiple molecular drugs [27, 28]. The generation of a population of cancer cells with stem-cell properties might provide another possible reason of resistance to EGFR inhibitor [29]. Common mechanisms of acquired resistance include secondary mutation in the target gene, activation of alternative pathway or feedback loop, and induction of EMT [23, 30]. Therefore, new therapy that concurrently attacks multiple critical pathways, inhibits the cross talk between diverse signals, and suppresses the CSC and EMT properties may be efficacious to overcome the resistance to molecular agents in EC.

Moreover, the administration of antiangiogenic agents, particularly antibodies against VEGF, leads to a more hypoxic tumor microenvironment [31], which enhances tumor cell invasion and metastasis by inducing the EMT- and CSC-like phenotype [32–34]. These works clearly suggest the need to combine antiangiogenic treatment in human tumors with new drugs targeting specific signaling pathways linked to the CSC/EMT phenotype. 

Another challenge is toxicity or the side effects associated with targeted therapies, such as harmful immune responses. These include “Off-target” adverse effects caused by a drug binding to an unexpected target and “On-target” adverse effects as a result of a drug binding to its intended target that is not only present in tumor cells, but also found in normal tissue [26]. 

## 4. Potential miRNA-Based Therapies in EC

Different from gene mutations, epigenetic changes that are associated with global gene regulation such as chromatin remodeling open a new field of cancer research [35]. Epigenetic silencing of tumor suppressor genes or epigenetic activation of oncogenes plays the important roles in the promotion of carcinogenesis and tumor progression [35]. Two common epigenetic changes are methylation at the promoter region and histone acetylation, which can be modulated using inhibitors of DNA methyltransferase (DNMT) and histone deacetylase (HDAC), respectively. Tumor suppressor genes including *PTEN* [36], DNA mismatch repair gene *hMLH1* [37], adenomatous polyposis coli (APC) [38], RAS-associated domain family member protein 1 (*RASSF1A*) [39], and *E-cadherin* [40] are more frequently silenced in type I tumor than in type II tumor. DNMT and HDAC inhibitors are already in clinical use for myelodysplasia and cutaneous T-cell lymphoma [41, 42]. Preclinical study has shown that DNMT and HDAC inhibitors induce cell apoptosis and suppress the growth of EC* in vivo* [43]. The combination of epigenetic modifiers with chemotherapy, hormonal therapy, and targeted therapy, has been proposed [44], and this may achieve better effect than single epigenetic agent for the treatment of EC. 

Another important mechanism for epigenetic regulation of gene expression is involved in noncoding RNAs, specifically small regulatory microRNA (miRNA). MiRNAs posttranscriptionally control gene expression by base pairing with the 3′ untranslated region of target mRNAs, which triggers either mRNA translation repression or RNA degradation [45]. 

As miRNAs are able to bind to their mRNA targets with either perfect or imperfect complementary, one miRNA may possibly have multiple target genes and concurrently influence different cellular signaling pathways [45]. Some miRNAs can function as either promoter or suppressor participating in a wide variety of biological functions of tumor, including cell proliferation, differentiation, migration, apoptosis, and recently EMT/cancer-stem-cell-like features [46]. Therefore, modulation of dysregulated miRNAs could be a powerful tool to correct abnormal signaling pathways related to EC. 

Altered expression profiles of microRNA have been observed in EC compared with normal endometrium [47]. Several miRNAs are differentially expressed between endometrioid and serous papillary EC, indicating that they could infer mechanisms that are specific to individual tumor subtypes [48]. Among those miRNAs elevated in endometrioid EC, the expression of miR-7 can be downregulated by using anti-miRNA oligonucleotides, leading to repressed migration and invasion of EC cells [49]. On the other hand, the level of miR-194 was significantly lower in EC patients with more advanced stage, and lower expression of this miRNA was associated with worse survival [50]. We found that overexpression of miR-194 by transfection with pre-miRNA molecule inhibited EMT phenotype and EC cell invasion by targeting the oncogene *BMI-1* [51]. We also identified miR-130b as one of the mutant p53-responsive 23 miRNAs, which is decreased in EC relative to adjacent normal tissue and directly targets the key EMT promoter gene *ZEB1* to revert p53-mutations-induced EMT features of EC cells [52]. MiRNAs are stable in various tissues and bodily fluids [53]. This property greatly facilitates the delivery of miRNAs to recipient cells via the blood or other compartments. Collectively, targeting those miRNAs that are deeply involved in EC progression would provide a promising therapeutic option for EC. 

**Figure 3:**
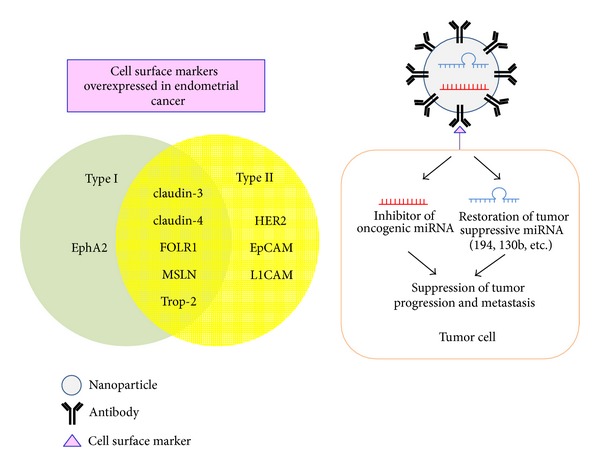
Potential miRNA-based therapies in EC. The use of antibodies against cell surface markers overexpressed in EC tissue might deliver targeted drugs to EC cells more specifically with fewer side effects on normal tissue. The nanotechnology can be used to develop a more effective delivery system for targeted agents, especially miRNA that might simultaneously modulate multiple signal pathways necessary for malignant phenotype of EC.

Forced expression of tumor suppressor miRNA and suppression of oncogenic miRNA are two strategies to achieve the goal of miRNA-based cancer treatment (Figure 3). Although previous results demonstrated that restoration of tumor suppressor miR-152 effectively inhibited EC cell growth *in vitro *and *in vivo *[54], obvious challenges of obtaining efficient delivery systems and tumor cell specificity must be resolved to allow clinical implementation.

The biochemical similarity between miRNA and siRNA suggests that the same delivery reagents developed for use with siRNA could be applied to the delivery of miRNA [55, 56]. Many efforts have been made to develop more effective and stable delivery systems [57]. Among them, nanoparticles confer greater miRNA stability, and the conjugation of nanoparticles to antibodies or cancer-specific ligands can notably improve their interactions with cancer cells [57]. By using the modification of GC4 single-chain fragment (a tumor-targeting human monoclonal antibody), nanoparticles injected intravenously showed greater accumulation in the tumor nodules rather than in liver and kidney. Moreover, the codelivery of three siRNAs together with miR-34a resulted in a more significant inhibition (80%) of metastatic melanoma than that obtained with siRNAs or miRNA alone [58]. These data demonstrate that the use of antibody targeting cell surface marker allows a selective delivery of miRNA into the tumor, and the combination of siRNA and miRNA could additively inhibit tumor growth and metastasis. 

As mentioned, another major issue for molecular cancer therapy is toxicity. To avoid potential side effects on normal tissue, increasing attention has been directed to the identification of tumor-specific surface markers including receptors and epitopes that are highly expressed in cancer cells, but not or minimally expressed in normal cells. Some potential tumor cell surface markers overexpressed in EC compared with normal endometrium might be used for targeted therapy (Figure 3). 

Eph receptor tyrosine kinases and their ephrin ligands influence central nervous system development, stem cell niches, and cancer cells [59]. Upon the binding of EphrinA1, the EphA2 receptor becomes tyrosine phosphorylated and interacts with several proteins to elicit downstream signaling, which regulate cell adhesion, proliferation, migration, and angiogenesis [60]. Overexpression of EphA2 was found in a high proportion of endometrioid EC and correlated with advanced disease and poor prognosis, whereas its expression is present at low levels in benign endometrial tissue [61]. The microtubule inhibitor conjugated to EphA2 antibody was shown to be specifically internalized by EphA2-positive EC cells, resulting in significant growth inhibition of EC cells both* in vitro *and *in vivo* [62]. 

The tight junction proteins claudin-3 and claudin-4 are highly expressed in endometrioid, serous papillary, and clear-cell EC [63], but less frequently found in normal endometrium [64]. Importantly, the intratumoral injection of cytotoxic *Clostridium perfringens* enterotoxin (CPE) that interacts with claudin-3 and claudin-4 in subcutaneous serous EC xenografts led to tumor disappearance and extended survival of animals [65], indicating that targeting claudin-3 and claudin-4 by CPE or other targeted treatment may efficiently suppress the progression of EC. 

Folate receptor alpha (FOLR1, a membrane-bound molecule) and mesothelin (MSLN, a glycosyl-phosphatidylinositol-linked cell surface antigen) that are upregulated in ovarian carcinoma [66] are also upregulated in serous EC more frequently than in endometrioid EC [67]. The expression of FOLR1 cannot be observed in normal endometrium tissue [67], suggesting that FOLR1 may serve as a good tumor cell surface marker for targeted therapy, and antibodies against FOLR1 may facilitate tumor-specific cellular uptake of molecular drugs. 

Trophoblast cell surface marker (Trop-2, a cell surface glycoprotein) is often overexpressed in various late stage epithelial tumor types with low or no expression in normal tissues [68]. Trop-2 is highly expressed in serous [69] and endometrioid EC [70]. Serous EC cell lines overexpressing Trop-2 show increased sensitivity to immunotherapy with hRS7, a humanized anti-Trop-2 monoclonal antibody [69]. Thus, Trop-2 would be an attractive target for EC immunotherapy. 

Epithelial cell adhesion molecule (EpCAM) is overexpressed on malignant cells from a variety of different tumors and is considered as a reliable marker for tumor-initiating cells [71]. The cell surface expression of EpCAM is significantly higher among serous EC specimen compared to in normal endometrial tissue [72]. Serous EC cell lines that are positive for EpCAM exhibit high sensitivity to EpCAM antibody-mediated cytotoxicity, suggesting that EpCAM may represent a novel therapeutic target for serous EC. 

In normal epithelium, the expression of L1 cell adhesion molecule (L1CAM) is undetectable. However, overexpression of L1CAM has been reported in many types of carcinomas [73]. L1CAM has been defined as a key driver for tumor cell invasion and EMT [73]. Of interest, L1CAM was absent in normal endometrium and the vast majority of endometrioid EC, but it was strongly expressed in serous and clear-cell EC [74]. The combined treatment with L1CAM antibodies and chemotherapeutic drugs in pancreatic and ovarian carcinoma model systems *in vivo* reduced tumor growth more efficiently than treatment with the cytostatic drug alone [75], indicating the value of L1CAM as a target for chemosensitizer in anticancer therapy for aggressive EC. 

Taken together, antibodies against various tumor cell surface markers would provide a possibility of delivering drugs to EC cells, with fewer side effects on normal tissue. The nanotechnology or other approaches might be used to develop a more effective delivery system for targeted drugs, especially miRNAs that might simultaneously modulate a broad range of gene networks necessary for malignant phenotype of EC.

## 5. Targeting the CSC/EMT Signaling Pathways in EC

CSC is defined as a rare population having the ability to self-renew, initiate tumor growth, and give rise to the heterogeneous tumor cell mass [76]. Growing lines of evidence suggest that CSCs do exist and support tumor maintenance during tumor formation [77]. CSCs of EC might be located in the basal layer of endometrium and are responsible for production of EC cells [78]. Sorted CD133 (+) subpopulations from EC cell expressed higher levels of oncogene *BMI-1* [51] and showed more aggressive potential and increased tumorigenicity in nude mice than CD133 (−) cells [79]. Stem-like cell subpopulations, referred to as “side population” (SP) cells, have been isolated from EC tissue and show self-renewal capacity and enhanced tumorigenicity *in vivo* [80]. Therefore, these results suggest that selective killing of such CSCs is an appealing therapeutic prospect for EC. 

**Figure 4:**
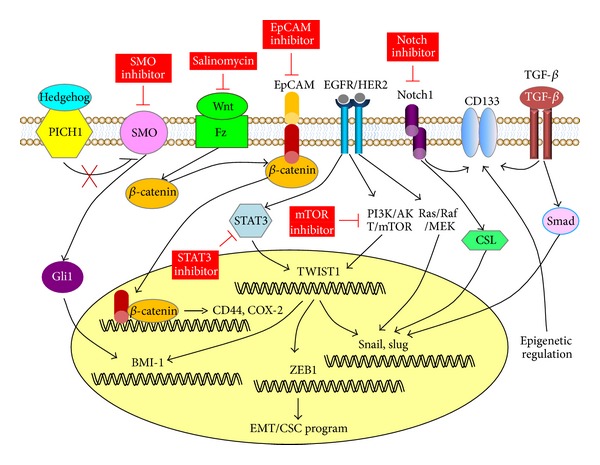
Targeting the CSC/EMT signaling pathways in EC. Tumor cells that undergo EMT not only increase their invasion ability, but also concurrently acquire cancer stem cell (CSC) properties. On the other hand, CSCs are associated with enhanced capacity to metastasize. At a molecular level, several signaling pathways involved in the self-renewal of CSCs, including Wnt/*β*-catenin, Hedgehog, and Notch signaling, can also induce EMT programs. Specific inhibitors targeting these CSC and EMT pathways efficiently suppress the malignant phenotype of EC cells. Other potential therapeutic candidates for EC treatment include Stattic (inhibitor of STAT3), Rapamycin (mTOR inhibitor), and CD133.

Tumor cells that undergo EMT can increase their invasion ability and concurrently acquire CSC properties [81, 82]. Indeed, CSC fractions within pancreatic cancer [83] and colon cancer [84] are associated with enhanced capacity to metastasize, a process that requires considerable invasive capacity. At a molecular level, these findings are consistent with the fact that several signaling pathways involved in the self-renewal of CSCs, including Wnt/*β*-catenin, Hedgehog (Hh), and Notch signaling [85], can also induce EMT programs [86] (Figure 4), supporting a molecular link between EMT and CSC program in human tumor [87]. Therefore, development of specific therapies targeted at these CSC and EMT pathways raises a hope for eliminating recurrent and metastatic disease and for improvement of patient survival. 

In malignant human mammary stem cells, activation of Hh signal components (SMO, PTCH1, and Gli1) increases the expression of downstream transcription factor BMI-1 and plays an important role in regulating stem cell self-renewal [88]. The overexpression of Hh-signal-related molecules is detected in EC tissue and involved in stimulated proliferation of EC cells [89]. In the same study, cyclopamine (a specific inhibitor of the SMO) has been shown to efficiently suppress the growth of EC cells [89]. 

Activation of Wnt/*β*-catenin pathway represented by the nuclear staining of *β*-catenin was shown to be more commonly detected in type I than type II EC [12]. More recent evidence suggests that gene sets indicating activation of Hh and Wnt/*β*-catenin signaling closely correlate with more aggressive EC and worse survival [90]. Wnt/*β*-catenin signaling was shown to induce the expression of downstream targets EpCAM and CD44 in hepatocellular carcinoma and EC, respectively [91, 92]. Salinomycin, a selective inhibitor of breast CSCs [93], was shown to induce apoptosis, inhibit Wnt/*β*-catenin signaling, and therefore repress the proliferation, migration, invasiveness, and tumorigenicity of SP cells obtained from invasive EC cells [94]. Thus, it is important to determine whether salinomycin alone, or in combination with other agents such as EpCAM-specific monoclonal antibody, could effectively induce apoptosis in CSC-like EC cells. 

High expression of Notch1 has been detected in EC patients with poor prognosis, and treatment with a reported Notch inhibitor DAPT [95] suppresses invasiveness of EC cells [96]. 

Other potential therapeutic candidates for EC treatment might include Stattic, Rapamycin, and CD133. Signal transducer and activator of transcription 3 (STAT3) has been shown to transcriptionally activate the expression of EMT inducer TWIST1, resulting in promoted oncogenic properties in breast cancer [97]. Stattic (an inhibitor of STAT3) can suppress EGF-enhanced invasive behavior of EC cells [98]. Rapamycin (an mTOR inhibitor) has been used to counter the effects of *PTEN* deletion and inhibit the development of leukemia-initiating cells while preserving normal stem cell populations [99]. Targeting CD133 (+) cells by CD133 antibody-cytotoxic drug conjugates effectively inhibits the growth of hepatocellular and gastric cancer cells *in vivo* and *in vitro* [100]. 

The most obvious concern is whether a therapy can selectively target CSC, but not destroy normal stem cell that could share many characteristics as CSC, such as the ability to self-renew and differentiate. However, CSCs and normal stem cells display different biological behaviors, mainly due to aberrant activation of several pathways involved in proliferation, self-renewal, differentiation, and metabolism in CSCs [101, 102]. Therefore, exploiting these molecular differences could be helpful to specifically target CSCs while preserving normal stem cells. Furthermore, the combined inhibition of Hh and EGFR signaling through the use of specific inhibitors can lead to the increased rate of apoptotic death and decreased invasiveness of prostate cancer cells [103], suggesting that this treatment might be affecting the CSCs. 

## 6. Targeting Immunosuppressive Molecular Pathways in EC

**Figure 5:**
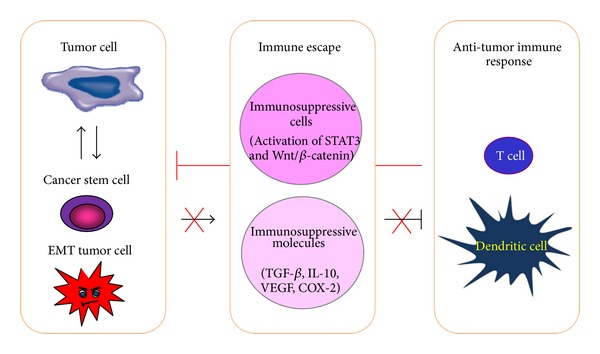
Targeting immunosuppressive molecular pathways in EC. Tumor cell induces immunosuppression by the production of immunosuppressive factors such as TGF-*β*, IL-10, VEGF, and COX-2. Tumor cells undergoing EMT can acquire both aggressive and immunosuppressive properties. Wnt/*β*-catenin pathway and STAT3-related pathway are activated in tumor cells and immunosuppressive cells and therefore they seem to be attractive targets for EC immunotherapy.

ECs are immunogenic tumors [104], and they mount potent antitumor immune responses, which might be ineffective at rejecting tumor, but might be potentially harnessed therapeutically [105]. Immune escape has been considered as the major malignant features of tumor cells. Several mechanisms are responsible for tumor immune escape, including the failure to recognize tumor cells by the immune system due to reduced major histocompatibility complex class I (MHC-I) expression, immunosuppression caused by tumor-cell-released immunosuppressive factors such as TGF-*β*, interleukin (IL)-10, VEGF, and cyclooxygenase-2 (COX-2), and immunoresistance resulting from the induction of EMT/CSC [104, 106, 107]. These data indicate that in addition to direct tumor cell killing, new targeted therapy might be also designed to reactivate the body's immune response against tumor cells (Figure 5).

Tumor stem cells (CD133+) have been shown to express low levels of MHC-I; however, the percentage of CD133-positive CSCs that expressed MHC-I can be significantly increased by the treatment with interferon-gamma [108], suggesting the possible use of MHC-I to generate anti-CSC immunity for human tumor including EC [106]. 

Some signal pathways that are activated in tumor cells are also dysregulated in immunosuppressive cells in cancer microenvironment. Immunosuppressive molecules released by tumor cells can activate STAT3 in immune cells, leading to tumour-induced immunosuppression [109]. In gastric cancer cells, oncogenic Wnt/*β*-catenin pathways enhance the transcription of COX-2, an immunosuppressive molecule [110]. Importantly, COX-2 is upregulated and associated with VEGF expression in EC tissue [111], and selective COX-2 inhibitor etodolac exhibits antiproliferative effects on EC tissue [112], indicating that targeting COX-2 may boost immune responses towards EC and repress EC progression [113]. Although the adverse effects on normal immune cells should be avoided, targeting STAT3 or Wnt/*β*-catenin pathway by specific inhibitor in tumor cells and immunosuppressive cells, or along with other immunotherapy, might restore the immunocompetence of EC patients.

## 7. Conclusion

Currently, targeted therapies have not entered clinical practice, and clinical trials involving genetic biomarkers (mTOR, HER2, EGFR, and VEGF) administered to ECs only resulted in modest effects. Therapy targeting epigenetic regulatory mechanisms such as miRNA will need to be developed to achieve a broader impact on multiple signal pathways necessary for EC development. The use of targeted cancer therapy remains challenging because of the lack of specificity for cancer cells. Targeted agents that are specific to cell surface markers overexpressed in tumor cells would avoid potential side effects on normal tissue. More importantly, we expect that new targeted therapies that specifically attack both cancer cells and CSC-like cells can be used together with immunotherapy that stimulates a host's immune response and with other traditional treatments to achieve better clinical prognosis of EC patients in the near future.
